# Integrating Telemedicine and Embodied Virtual Reality: A Novel Functional Training Intervention for Chronic Shoulder Pain

**DOI:** 10.1177/29941520251381338

**Published:** 2025-10-13

**Authors:** Kristine Beebe, Anthony Alvarez, Lincoln Nguyen, James Petros, Michael Trujillo, Oytun Aygün

**Affiliations:** ^1^ Karuna Labs. Inc., San Francisco, California, USA.; ^2^ Allied Pain and Spine, Los Gatos, California, USA.

**Keywords:** digital therapeutics, functional rehabilitation, chronic pain, telehealth, telemedicine

## Abstract

Chronic shoulder pain is a common musculoskeletal pain condition that can have a profound impact on a sufferer’s life and a large socioeconomic health care burden to society. The aim of this study was to investigate the effects of a functional rehabilitation therapy using virtual embodiment in VR, on chronic shoulder pain, delivered in a telemedicine format. Ten chronic shoulder pain patients were recruited from local pain clinics. Patients engaged in three rehabilitation sessions per week over the course of 8 weeks. Rehabilitation sessions were delivered using an Oculus Quest head-mounted display and hand controllers. Rehabilitation exercises (virtual embodiment training) leverage techniques from graded motor imagery, such as visual mirror feedback and augmentation of the virtual avatar’s movements. Eight assessments on function and psychological aspects relevant to chronic pain were measured prior to the first session, at week 4, and at the end of 8 weeks in order to investigate the effect of the virtual embodiment training on the symptoms of chronic shoulder pain. Range of motion was measured within the virtual environment at each exercise session for shoulder flexion, scaption, and abduction. A one-way repeated measure analysis of variance revealed that virtual embodiment training improved symptoms of disability measured by the disabilities of the arm, shoulder and hand questionnaire (F(2, 9) = 8.054, *p* = 0.004). Virtual embodiment training improved function as measured by upper extremity functional index (F(2,9) = 6.506, *p* = 0.009), pain catastrophizing scale (F (2, 9) = 5.792, *p* = 0.013), fear avoidance beliefs questionnaire (F(2, 9) = 6.722, *p* = 0.008), pain self-efficacy questionnaire (F (2, 9) = 4.841, *p* = 0.023) and yellow flag risk form (F(2,9) = 6.563, *p* = 0.008). No significant effect was observed for the SF-36 questionnaire of quality of life, or the Tampa Scale of Kinesiophobia. These results indicate that telemedicine-enabled functional rehabilitation for chronic shoulder pain may improve chronic pain symptoms such as disability, function, pain catastrophizing, and fear avoidance in chronic pain patients.

## Introduction

The emergence of digital therapeutics offers a promising avenue to expand and improve the prevention and treatment of a multitude of diseases. Therapeutics in the form of software for computers, mobile devices, and technologies such as virtual reality (VR) have the potential to expand access to care and offer noninvasive, nonpharmacological, and nonaddictive methods to treating conditions such as chronic pain. Defined as pain with a continual duration greater than 6 months,^1^ chronic pain is a debilitating condition that can be treatment-resistant.^2^ While existing therapies such as nonsteroidal anti-inflammatories, physical therapy, implanted spinal cord stimulators, and transcutaneous electrical nerve stimulators are often prescribed, their efficacy is often inadequate and temporary. Opioids are commonly prescribed due to demonstrated effectiveness for acute pain, however, opioids are largely ineffective for chronic pain and come with inherent risk of addiction and overdose.^3^

In this context, more efficient treatment options are necessary for managing and treating chronic pain. One of the most effective treatment strategies up to date is functional restoration programs that combine traditional treatments with biofeedback, pain psychology, and cognitive behavior therapy in an intensive inpatient setting, for example, 6 h/day for up to 5 weeks.^4^ While functional restoration programs are promising, they require a large time commitment, are expensive, and not widely accessible. Recently, delivering therapies and treatment in VR has demonstrated effectiveness for chronic pain.^5–8^ Rapid improvements in VR technologies offer a promising opportunity to expand treatment accessibility for patients with chronic pain because patients can receive therapies and treatments from their home. Meanwhile, the COVID-19 global pandemic has elucidated the need to leverage telemedicine for the treatment of chronic pain.^9^ Telemedicine is the practice of delivering care to patients remotely over the internet using technologies such as smartphones and personal computers. Various therapies, such as psychotherapy and cognitive behavioral therapy, which have been shown to be effective treatments for chronic pain,^10–12^ lend themselves well to a telemedicine approach. Other treatments, such as physical therapy, may be delivered remotely in patients’ homes if technology can be developed that incorporates techniques and principles used in the clinic. Physical therapy delivered in VR has been shown to be effective for pediatric burn victims,^13^ and people suffering from Parkinson’s disease, with VR demonstrating superiority over conventional physical therapy.^14^

Chronic pain is described as a biopsychosocial phenomenon resulting from a combination of psychological and physical factors, in the presence or absence of an identified pathology that increases the sensitivity of the nervous system to pain.^15^ Maladaptive plasticity in the central nervous system has been demonstrated in response to persistent pain, where synaptic plasticity occurs in sensory pathways that are active in the presence of injury and pain.^16,17^ Specifically, long-term potentiation in the anterior cingulate cortex has been proposed as a significant mechanism leading to a chronic pain state where pain thresholds are shifted due to heightened response of the brain region that is active when anticipating painful stimuli. Distraction during painful states modulates activation within the anterior cingulate cortex relative to a painful state without distraction.^18^ A proposed mechanism of the analgesic effects of VR is alteration of activity of the anterior cingulate cortex, a region of the brain that is involved in attentional processes related to pain perception.^19^ In addition, VR hardware has demonstrated accurate motion tracking capabilities, affording the ability to deliver functional exercises and track performance.^20^ In addition, the use of VR has been shown to reduce pain and depression symptoms in patients with fibromyalgia while also adding an increase in positive affect, the experience of positive emotions.^21^ Immersive VR has demonstrated improvements in subjective pain relief and improvements in daily function in patients suffering from upper limb complex regional pain syndrome.^22^ In patients with phantom limb pain, immersive VR decreased phantom limb pain and elicited the transfer of sensation of the amputated limb.^23^

The present study sought to determine the effects of delivering functional rehabilitation through virtual embodiment for patients with chronic shoulder pain in a telemedicine format. Virtual embodiment is a feature of VR where visual feedback from a virtual avatar elicits the perception that the position and movements of the virtual avatar occur in the first-person, resulting in ownership and control over the virtual avatar. Virtual embodiment training leverages virtual embodiment to deliver exercises that require functional movements to complete tasks in VR. For example, a floating flower is positioned in front of the user’s field of view in the virtual environment, as the goal of the exercise is to grasp the flower using the arm and hand of a virtual avatar. As the user moves a hand controller with their anatomical hand, their virtual avatar matches the movement in the virtual space, providing visual feedback of the corresponding movements of the virtual avatar. The flower is positioned in an area in a virtual environment that requires shoulder flexion, scaption, or abduction to successfully grasp the flower. The exercise is repeated for several minutes to promote multiple repetitions at each plane of movement. The present study sought to gain insight into whether immersive VR for functional rehabilitation may improve symptoms of chronic pain, such as shoulder range of motion, disability, and chronic pain-related psychological outcomes.

## Material and Methods

### Ethical considerations

This study protocol was conducted in accordance with the ethical standards of the Declaration of Helsinki and approved by ADVARRA (reference number: Pro00026459), an independent institutional review board. This trial was registered at ClinicalTrials.gov (NCT04060875). All patients provided written informed consent prior to participating in the study.

### Participants

**FIG. 1.**
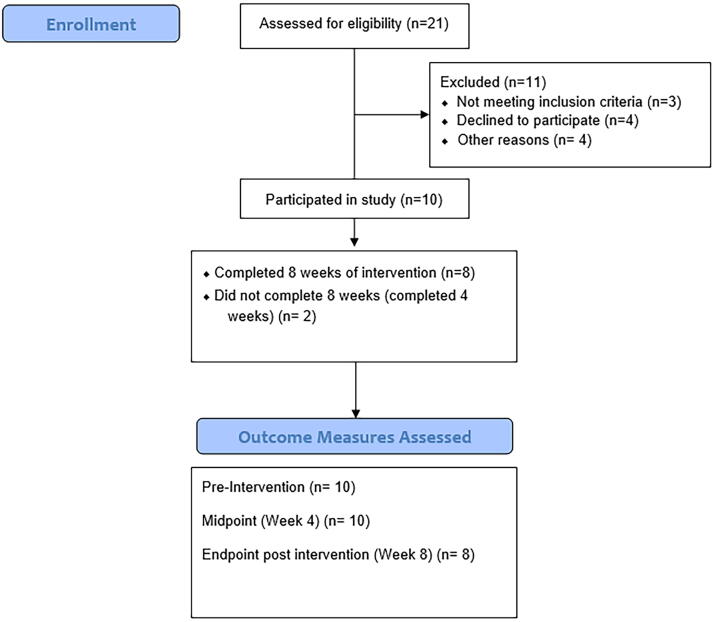
A consort diagram of patient process through the study.

Ten adult chronic pain patients (seven Female, three Male, mean age = 48.44 years) participated in this study. Eight patients presented with chronic pain in the left shoulder, and two presented with right chronic shoulder pain. Patients were recruited from a local pain clinic. A total of 21 patients were assessed for eligibility. Patients were excluded if they reported a history of motion sickness, a history of seizures, or cognitive impairments. Two patients were excluded prior to participation for a history of vertigo or motion sickness, and one was excluded due to color blindness. Four patients declined to participate prior to beginning treatment. Four patients were excluded due to impending surgeries scheduled that would prevent them from completing the study protocol (see Fig. 1 consort diagram). Among the 10 participants, eight patients completed the 8-week protocol, and two patients completed 4 weeks.

### Intervention

Patients who volunteered to participate in the study were shipped an Oculus Quest six-degrees-of-freedom wireless head-mounted VR display (HMD) and hand controllers. Patients were assigned a licensed health coach by the NHCI NBHWC certification exam, all coaches had previously followed a 3-month neuroscience of pain training to assist with the use of the VR hardware. The health coach met with the patients via an HIPAA-compliant telehealth video chat platform (Practice Better; https://practicebetter.io/). Patients were instructed on the safe use of the VR headset and hand controllers and instructed on how to create the safe play guardian of the Oculus Quest VR system and how to connect the HMD to a home wi-fi. After the patients confirmed that they were comfortable using the Oculus Quest, they were advised to complete three sessions of functional rehabilitation in VR exercises per week over the course of 8 weeks. All functional rehabilitation exercises were self-administered by each patient. The coach conducted telehealth video chat sessions with each patient once per week and administered a structured curriculum that included pain neuroscience education, goal setting, instructions for pacing and grading, and flare-up management.

Functional rehabilitation exercises were delivered through KarunaHOME (Karuna Labs, Inc., San Francisco, CA). KarunaHOME is a VR software delivered on the Oculus Quest to provide rehabilitation exercises in patients’ homes. KarunaHOME Virtual Embodiment Training™ consists of five functional rehabilitation exercises designed on the principles of graded exposure and mirror therapy.

**FIG. 2.**
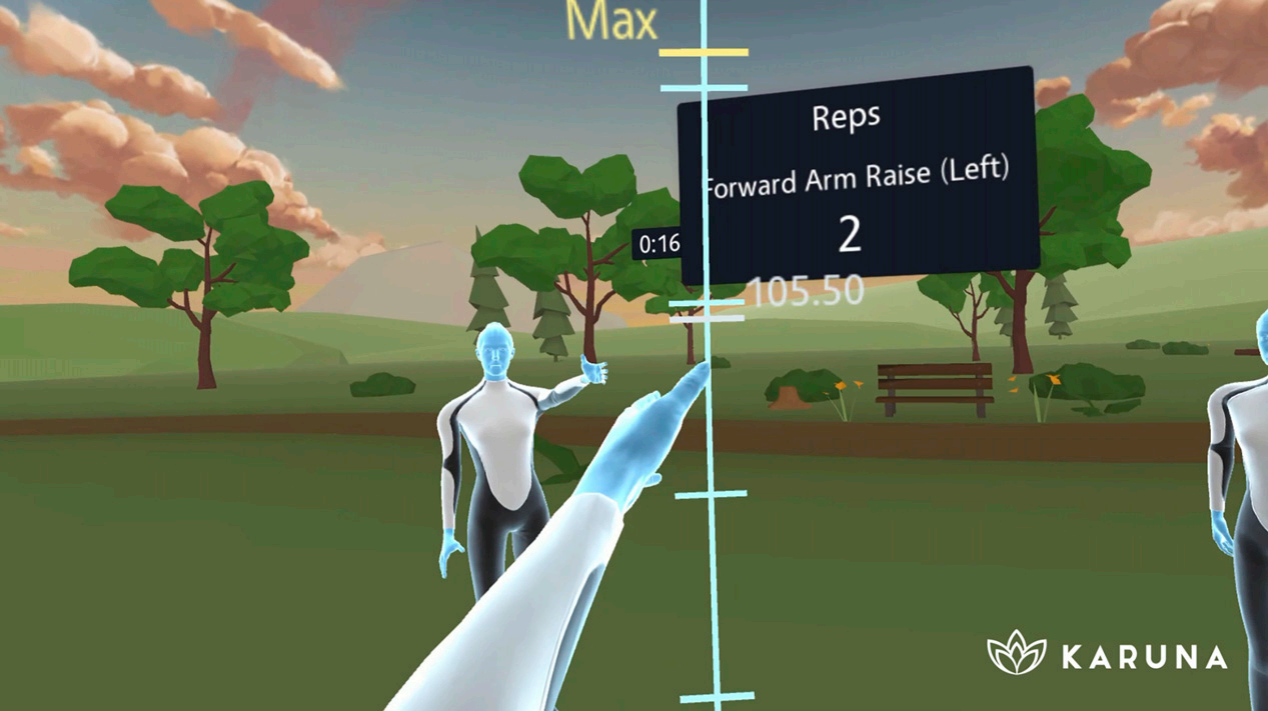
A first-person view of the calibration exercise. The patient sees their embodied avatar, and as they perform the movement.

Each session of functional rehabilitation began with a calibration exercise that measured shoulder range of motion for flexion, scaption, and abduction (Fig. 2). The calibration exercise progressed through shoulder flexion, shoulder scaption, and shoulder abduction one at a time, on each side. The patient was instructed in VR to perform three repetitions within a comfortable range. The second exercise was a functional movement exercise that promoted shoulder flexion, scaption, and abduction while engaged in a reach and grasp task. Patients reached for an orb in the form of a floating lotus flower positioned in front of the patient in the virtual environment. Patients grasped the orb using the trigger of the Oculus Quest hand controller and then tossed the orb into a pond by producing either an overhand or underhand throwing motion and releasing the trigger. The second exercise also incorporated a mirror visual feedback (MVF) manipulation where the avatar’s contralateral limb moved relative to the patient’s movement. For example, if the patient moved their right arm, they would see the embodied avatar’s left arm moving. MVF was used for half the duration of each exercise. The third exercise was a reach and grasp exercise where patients reached to grasp colorful flowers in front of their embodied virtual avatar. The goal of this exercise was to match flowers of the same color in sets of three or more. The patient reached up for a flower to remove it, and then the flowers above fell into place. The exercise required patients to reach a maximum height by continuing to remove flowers. The fourth exercise was a painting exercise designed to promote complex, dynamic shoulder motion. The goal of this exercise was to connect numbered dots to create different shapes in the sky by painting with a virtual paintbrush. The first few shapes were directly in front of the patient and were simple movements such as a circle, a triangle, and a heart. The shapes became progressively more difficult, requiring greater shoulder range of motion for reaching above and to the side, and progressed to more complex shapes to connect. The final exercise was a simulated activity of daily living in the form of a bow-and-arrow experience. Movements were designed to mimic motions that are included in activities of daily living, such as putting on a seatbelt or shampooing hair. There were three distinct exercises within the bow and arrow activity. The first exercise required patients to reach the affected arm across the body (horizontal adduction) to the quiver on the opposite hip to grasp an arrow and then bring the arrow to the bow (shoulder flexion) and pull back (scapular retraction), and shoot towards a target. The second exercise required patients to reach with the affected arm over and behind the opposite shoulder to the quiver to get an arrow and then bring the arrow to the bow (shoulder flexion) and pull back (scapular retraction), and shoot. The third exercise required patients to reach over the affected shoulder (shoulder flexion, abduction, and internal and external rotation). Each session progressed from the first exercise to the last exercise with 1 min of a guided breathing exercise in between each exercise. Patients completed three sessions per week over the course of 8 weeks.

### Measurements

To assess the effects of VR telehealth on delivered functional rehabilitation, a battery of pain assessments was administered at three timepoints (pre, mid, and post), prior to beginning the 8-week protocol (pre), at the midpoint, that is, at week 4 (mid), and after completing the 8-week protocol (post). The disability of the arm, shoulder, and hand (DASH) questionnaire was used to assess shoulder disability.^24^ The upper extremity functional index (UEFI) was used to assess shoulder function and the functional disability of the upper limb.^25^ The 36-Item Short Form Survey (SF-36) was used to assess quality of life.^26^ The pain catastrophizing scale (PCS) was used to assess the psychological experience of pain, how people feel, and what they think about when they are in pain.^27^ The fear avoidance beliefs questionnaire (FABQ) physical activity subscale was used to assess fear of movement due to chronic pain.^28^ The Tampa Scale of Kinesiophobia (TSK) was used to assess fear of movement.^29^ The pain self-efficacy questionnaire (PSEQ) was used to assess patient confidence to do activities despite pain.^30^ The yellow flag risk form (YFRF) was used to assess psychosocial risks, such as depression, anxiety, fear avoidance, and pain catastrophizing.^31–33^ The FABQ and PCS are included in a protocol that is registered in clincaltrials.gov (NCT: NCT04060875).

### Statistical analysis

A one-way repeated measures analysis of variance (patients × timepoints) was used to assess statistical differences in outcome measures as a function of rehabilitation protocols delivered. Each outcome measure was repeated three times, a pre-test was assessed before beginning the KarunaHOME program, a mid-point assessment at the 4-week mark, and a post assessment after the completion of the 8-week program. A Tukey post-hoc test was used to assess pairwise comparisons of the three timepoints to determine if mid and post endpoints significantly differed from baseline (pre).

## Results

### Outcome measures

#### The disability of the arm, shoulder, and hand

**FIG. 3.**
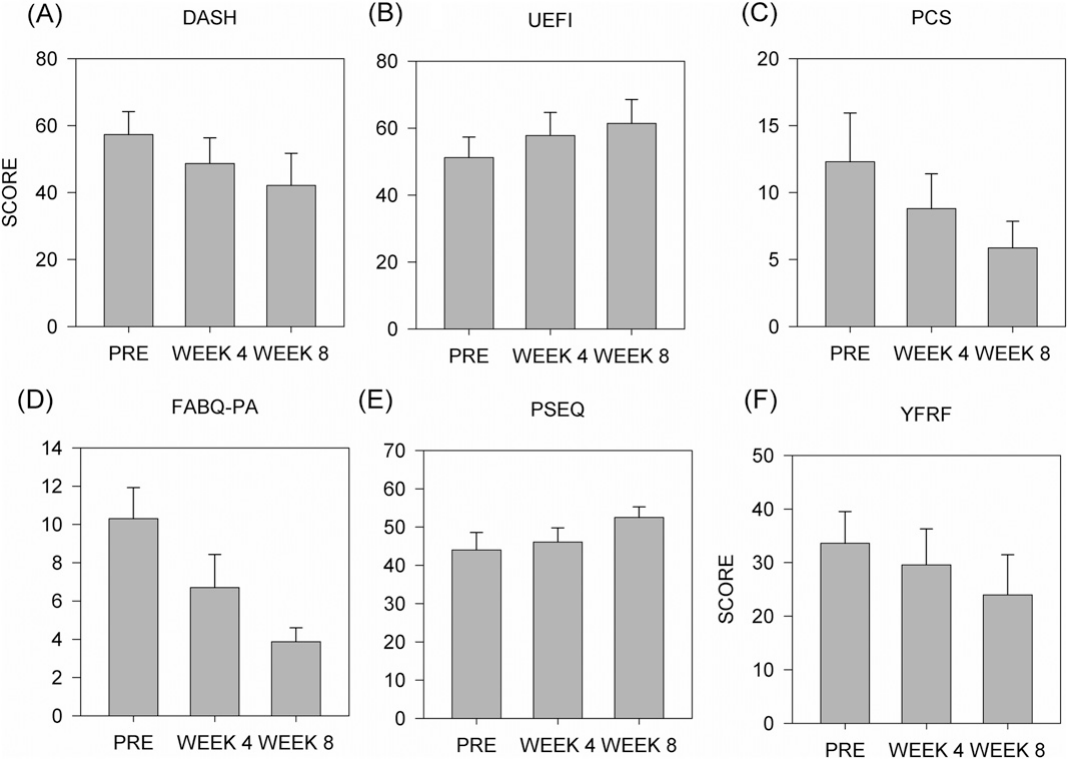
**(A)** Average scores for disability of the arm, shoulder, and hand questionnaire. **(B)** Average scores for upper extremity functional index. **(C)** Average scores for pain catastrophizing scale. **(D)** Average scores for the physical activity subscale of fear avoidance beliefs questionnaire. **(E)** Average scores for pain self-efficacy questionnaire. **(F)** Average scores for yellow flag risk form. The x-axes represent the timepoint at which questionnaires were administered. The y-axes represent the score for each measurement. Bars represent the average score and vertical lines represent the standard error measure.

Figure 3A represents the mean + SEM for DASH. A significant main effect was observed between outcome measure time points (F (2, 9) = 8.054, *p* = 0.004). A significant difference was observed between timepoints, revealing improvements in disability from pre to post (*p* = 0.003) and from mid to post (*p* = 0.036).

#### The upper extremity functional index

Figure 3B represents the mean + SEM for UEFI. A significant main effect was observed between outcome measure time points (F (2, 9) = 6.506, *p* = 0.009). A significant difference was observed between timepoints, revealing improvements in function from the pre to post (*p* = 0.007).

#### The pain catastrophizing scale

Figure 3C represents the mean +SEM for PCS. A significant main effect was observed between outcome measure timepoints (F (2, 9) = 5.792, *p* = 0.013). A significant difference was observed between timepoints, revealing improvements in pain catastrophizing from pre to post (*p* = 0.01).

#### The fear avoidance beliefs questionnaire

Figure 3D represents the mean + SEM for FABQ. A significant main effect was observed between outcome measure timepoints (F (2, 9) = 6.722, *p* = 0.008). A significant difference was observed between timepoints, revealing improvements in fear avoidance beliefs between pre and post (*p* = 0.007).

#### The pain self-efficacy questionnaire

Figure 3E represents the mean + SEM for PSEQ. A significant main effect was observed between outcome measure timepoints (F (2, 9) = 4.841, *p* = 0.023). A significant difference was observed between timepoints, revealing improvements in self-efficacy between pre and post (*p* = 0.03).

#### The yellow flag risk form

Figure 3F represents the mean + SEM for YFRF. A significant main effect was observed between outcome measure timepoints. (*F* (2,9) = 6.563, *p* = 0.008). A significant difference was observed between timepoints, revealing differences between pre and post (*p* = 0.006).

#### The 36-Item Short Form Survey

No significant effect was observed for the SF-36 (*F* (2,9) = 2.393, *p* = 0.123) measuring quality of life.

#### The Tampa scale of Kinesiophobia

No significant effect was observed for TSK (*F* (2,9) = 1.387, *p* = 0.278).

The results of this study are close to or exceed the minimal clinically important difference reported on several measures. On average, DASH improved by 4.015 points from pre to mid and 9.834 points from pre to post. A clinically important difference of 10.83 points has been reported for DASH.^34^ On average, the UEFI improved by 6.6 points from pre to mid and 10.175 points from pre to post. A clinically important difference of 9.4 has been reported for UEFI.^25^ On average, PCS improved by 30% from pre to mid and 61% from pre to post. A clinically important difference of a reduction in PCS score of 38% has been reported.^35^

## Discussion

This study aimed to assess the effects of a novel digital therapy for chronic shoulder pain patients, which delivers functional rehabilitation through virtual embodiment in VR in a telemedicine format. Participants demonstrated significant improvements across multiple outcome measures following the intervention. Participants reported reduced limitations in daily activities, as the disability of the arm that was injured decreased significantly over the course of the 8-week intervention, reflected in the DASH scores. The shoulder function also improved, as indicated by UEFI outcomes, showing enhanced mobility and usability. Pain catastrophizing levels were significantly reduced, as measured by PCS, highlighting a reduction in negative pain-related thoughts. Fear-avoidance beliefs, evaluated using FABQ, significantly declined, suggesting that participants became less apprehensive about physical activities. Self-efficacy improved substantially, with PSEQ results reflecting greater confidence in managing pain. In addition, other psychological outcomes, captured by YFRF, demonstrated significant progress from the beginning to the end of the intervention. However, no notable changes were observed in overall health-related quality of life or levels of Kinesiophobia, as assessed by the SF-36 and the TSK, respectively.

Treating persistent pain can be challenging due to changes in pain perception that may result from maladaptive neuroplasticity within the central nervous system that alters the processing of sensory input from the body.^36–40^ The results of the present study suggest that Virtual Embodiment Training™, delivered in a telemedicine format, may influence chronic pain mechanisms. Chronic pain is suggested to be a learned condition that is a complex biopsychosocial interaction where changes within the central nervous system alter the perception of pain, causing patients to experience pain in the absence of painful stimuli (allodynia) or experience pain as more intense (hyperalgesia).^41,42^ While there may be many mechanisms that result in chronic pain, maladaptive neuroplasticity is suggested to be the driving force in chronic pain.^17,40^ The use of VR therapies in treating pain, whether acute or chronic, is a relatively novel approach trying to leverage VR technology to influence the perception of pain.^7,13,43–49^ Two distinct approaches are suggested to elicit analgesic effects in VR.^13,44,50^ Distraction therapy, briefly diverting attention away from pain while users are engaged in a VR experience, and immersiveness. In this study, we used virtual embodiment, the phenomenon where a user is immersed in a virtual avatar and perceives actions of the virtual avatar as their own, to deliver functional rehabilitation in VR. While the patients controlled the movement of the virtual avatar, visual feedback from the avatar and the environment were manipulated to constrain task requirements so that functional shoulder motions were required to successfully complete a task. Although future studies are needed to investigate the specific neurological pathways involved in the influence of VR on chronic pain, the proposed mechanism of the current intervention is renormalization of associations between pain and movement through virtual embodiment that provides a recontextualization of associations between sensory feedback of movement due to altered visual feedback of movements of the virtual avatar.

Multiple psychological measures relevant to chronic pain conditions improved in the present study, suggesting that Virtual Embodiment Training™ combined with education and coaching may influence chronic pain through psychological mechanisms. The YERF scores allow investigating psychosocial risks; they are often used to sub-classify participants based on their scores of psychosocial risk in order to offer them adaptive treatment protocols.^33,51^ In the present study, YERF scores pain catastrophizing levels, and fear-avoidance beliefs, significantly reduced, suggesting that participants changed attitudes towards the experience of pain. Fear-avoidance of models of chronic pain suggests that attitudes about pain predict pain-related disability.^52–56^ Similarly, pain catastrophizing has been associated with more negative attitudes to medical interventions among chronic pain patients.^57,58^ There is evidence to support the proposed mechanism that virtual embodiment can promote the renormalization of the processing of movement-induced pain. In patients with chronic musculoskeletal pain, moving the affected joint or limb often results in pain. Due to the strong relationship between sensory and motor systems within the nervous system and the correlation between pain and movement in time, the perceptual association between movement and pain is strengthened in a Pavlovian classical conditioning context. This association leads to fear of movement because movement is associated with pain, in other words, fear-avoidance.^53,54,59^ In the imprecision hypothesis of chronic pain, the classical conditioning model is expanded with the hypothesis that pain-associated kinesthetic cues contribute to pain perception and that multisensory information in the form of temporal, spatial, and proprioception is not precisely processed within the central nervous system in people with chronic pain.^60^ There is support for the notion that altering sensory feedback in a VR environment influences function and the association between movement and pain. In chronic neck pain patients, the pain-free range of motion increases when visual feedback is understated in a VR environment and reduced when visual feedback is overstated.^61^ Interestingly, Harvie et al (2015) report that pain intensity did not differ across conditions when visual feedback was either understated, accurate, or overstated in VR, despite reporting differences in function as measured by range of motion. Those results support the imprecision hypothesis that pain can be influenced by sensory feedback and that altering sensory feedback in VR can influence function in patients with chronic pain. Our hypothesis that virtual embodiment can renormalize the processing of movement-induced pain is anchored in the hypothesis that chronic pain is influenced by associative learning between movement and multisensory processing. While not specifically measured in this study, we posit that the analgesic effects in VR are influenced by altering the visual feedback of the position and movement of a virtual avatar and that this can influence fear avoidance that arises through associative learning. The improvements we report on fear avoidance measured in this study support this. Furthermore, there is evidence in the literature that the results of this study may be explained by the ability of virtual embodiment to influence pain processing in the central nervous system. An fMRI study that compared immersive VR to opioid administration on pain ratings in an experimental pain condition reported that VR influenced pain-related brain activity and that the combination of VR and opioid administration had an additive benefit over opioid administration alone.^47^ Interestingly, activation in the anterior cingulate cortex decreased in the combination condition of VR and opioid together, but was not statistically significant in the VR or opioid conditions alone. The VR-only condition resulted in significant changes in activation in the insula, primary somatosensory cortex, secondary somatosensory cortex, and thalamus. This suggests that immersive VR can influence the central nervous system processing of painful stimuli. Therefore, we postulate that engaging in functional exercises in a virtual environment may have the ability to renormalize the association between movement and pain in chronic pain patients.

Among the functional measures, disability of the affected body part showed improvements already after 4 weeks of treatment, while the upper extremity functional index measure showed improvement after 8 weeks of treatment. The psychological measures, pain catastrophizing, fear avoidance beliefs, confidence in coping with ongoing pain, and pain-related psychological distress, showed improvement after 8 weeks, but not after 4 weeks. This may suggest that either 4 weeks of treatment is not sufficient for improving functional and psychological symptoms of chronic pain or that the instruments used to assess improvement are not sensitive enough to measure subtle improvements. Nonetheless, the improvements in disability, function, and multiple psychological outcomes after 8 weeks of treatment suggest that the use of VR to deliver functional rehabilitation is an apparent viable treatment method for multiple dimensions of chronic shoulder pain. Further studies are needed to determine the effects of frequency and duration of an intervention on different aspects of chronic pain. Finally, there is a growing body of evidence that highlights the potential of VR in chronic pain management. VR interventions for chronic pain have been associated with improvements in pain reduction, disability, and functional outcomes in various chronic pain conditions, with no to very little side effects associated with VR (e.g., occasional nausea).^62–64^ One study reports greater therapeutic effects when VR is combined with conventional rehabilitation compared to standard treatment alone.^63^ Our study adds to the evidence that VR and online formats of chronic pain treatment are feasible and effective. Further studies are needed to explore the mechanisms of action of such treatments for chronic pain, as these models may offer additional accessibility and adherence benefits, particularly in remote or restricted settings.

There are several other limitations to this study. Virtual embodiment training was delivered along with behavioral health coaching; therefore, we cannot conclude that the results were driven by virtual embodiment training alone. In addition, we did not quantify the functional components of virtual embodiment training, however, the exercises were designed to be functional in nature by including virtual activities of daily living and graded exposure. Additional research is needed to determine the generalizability of these study results to real-world scenarios. Finally, this study is a single-arm study without a comparison or control arm. A larger, controlled trial is needed to confirm the benefits of Virtual Embodiment Training™. Future research is needed to clarify optimal protocols and to compare the long-term outcomes of different delivery methods.

Finally, the application of VR in therapeutic interventions has been explored as a promising non-pharmacological approach for managing chronic pain. With the rapid evolution of VR technology, including the development of advanced headsets like the Oculus Rift (Meta) and HTC Vive, its potential in clinical settings continues to expand. Recent innovations in portable VR devices further reinforce the viability of VR-based treatments as effective alternatives for pain management. In the present study, the patients were able to self-administer functional rehabilitation exercises from their home using VR. This study provides support for the possibility that embodiment in VR can be used to deliver functional rehabilitation in the home for chronic pain patients. Furthermore, 8 weeks of virtual embodiment treatment may have positive benefits on the symptoms of chronic pain.

## Abbreviations Used

FABQ: Fear avoidance beliefs questionnaire
HMD: Head-mounted display
MVF: Mirror visual feedback
NBHWC: National Board for Health & Wellness Coaching
NHCI: National Health Information Center
PCS: Pain catastrophizing scale
PSEQ: Pain self-efficacy questionnaire
SF-36: 36-Item Short Form Health Survey
SEM: Standard error of the mean
TSK: Tampa Scale of Kinesiophobia
UEFI: Upper extremity functional index
VR: Virtual reality
YFRF: Yellow flag risk form
